# Risk Factors for Methicillin Resistant *Staphylococcus aureus*: A Multi-Laboratory Study

**DOI:** 10.1371/journal.pone.0089579

**Published:** 2014-02-26

**Authors:** Boudewijn Catry, Katrien Latour, Béatrice Jans, Stien Vandendriessche, Ragna Preal, Karl Mertens, Olivier Denis

**Affiliations:** 1 Healthcare-Associated Infections & Antimicrobial Resistance, Public Health & Surveillance, Scientific Institute of Public Health, Brussels (WIV-ISP), Belgium; 2 Laboratoire de Référence MRSA - Staphylocoques, Department of Microbiology, Hôpital Erasme, ULB, Brussels, Belgium; 3 Intermutualistic Agency (IMA-AIM), Brussels, Belgium; Huashan Hospital, Shanghai Medical College, Fudan University, China

## Abstract

**Background:**

The present study aimed to investigate the dose response relationship between the prescriptions of antimicrobial agents and infection/colonization with methicillin resistant *Staphylococcus aureus* (MRSA) taking additional factors like stay in a health care facility into account.

**Methods:**

Multi-centre retrospective study on a cohort of patients that underwent microbiological diagnostics in Belgium during 2005. The bacteriological results retrieved from 17 voluntary participating clinical laboratories were coupled with the individual antimicrobial consumption patterns (July 2004-December 2005) and other variables as provided by pooled data of health insurance funds. Multivariate analysis was used to identify risk factors for MRSA colonization/infection.

**Results:**

A total of 6844 patients of which 17.5% died in the year 2005, were included in a logistic regression model. More than 97% of MRSA was associated with infection (clinical samples), and only a minority with screening/colonization (1.59%). Factors (95% CI) significantly (p≤<0.01) associated with MRSA in the final multivariate model were: admission to a long term care settings (2.79–4.46); prescription of antibiotics via a hospital pharmacy (1.30–2.01); age 55+ years (3.32–5.63); age 15–54 years (1.23–2.16); and consumption of antimicrobial agent per DDD (defined daily dose) (1.25–1.40).

**Conclusions:**

The data demonstrated a direct dose-response relationship between MRSA and consumption of antimicrobial agents at the individual patient level of 25–40% increased risk per every single day. In addition the study indicated an involvement of specific healthcare settings and age in MRSA status.

## Background

In hospitals, antimicrobial resistance leads to increased healthcare costs primordially due to a higher morbidity and mortality from infectious diseases, and increased length of stay. This has been demonstrated, among other pathogens, for methicillin resistant *Staphylococcus aureus* (MRSA) [1]. In defined healthcare settings the relationship between antimicrobial consumption and MRSA is well established and was found to be dose-dependent [2], [3]. Colonisation with MRSA is associated with a 4-fold increase of infection [4]. Despite the established pathogenicity in community associated *S. aureus* strains [5], the relative contribution of antimicrobial consumption to antimicrobial resistance under different healthcare settings is complex and at the individual patient level this remains to be quantitatively assessed.

The objective of this multicentre retrospective cohort study was to investigate a dose-effect relationship between consumption of antimicrobial agents and MRSA infection/colonisation at the individual patient level, taken into account variables such as class of antimicrobials administered, age and type of healthcare setting.

## Materials and Methods

### Study Design

The microbiological results were retrieved from 17 voluntary participating clinical laboratories during 2005 in Belgium (convenience sample based upon willingness of participants). These bacteriological results were coupled with the individual antimicrobial consumption patterns during this observation period, and extended with a half year prior to the first laboratory observations (July 2004–December 2005). Briefly, national registry numbers were provided by the laboratories for each patient that underwent reimbursed bacteriological examinations. Via an encrypted key code, antimicrobial prescription records from the Belgian reimbursement agency (RIZIV-INAMI) were coupled. The Intermutualistic Agency (IMA-AIM) served as third trusted party (TTP). For every patient, only the first *S. aureus* isolate (infection/colonization) was retained to minimize confounding through underlying disease and/or severity of infection.

### Laboratory Results

Data collected included a unique patient identification number, sample date, sample site (matrix), identification up to (sub)species level, and antibiogram. The latter susceptibility testing results were mainly done by the Kirby Bauer disk diffusion technique according to CLSI guidelines (Clinical and Laboratory Standards Institute, at that time NCCLS), which was often performed with semi-automated systems (e.g. SIRScan). Modifications were present according to the manufacturer for deviations in disk charge or diameter. The majority of Belgian hospitals worked with Neosensitabs (Rosco, Taarstrup, Denmark) for producing these antibiograms. The detection of *S. aureus* was assumed to be done according to the laboratory internal routine methodology, and the definition of MRSA versus methicillin susceptible *S. aureus* (MSSA) was based on the susceptibility testing result for oxacillin (cefoxitin). All participating labs were at the time of survey certified by a mandatory external quality control organisation (Vernelen K, WIV-ISP, personal communication). For the purposes of this study, infection was attributed if microbiological results were obtained from clinical samples, i.e. not categorized by the laboratory as: ‘surveillance’; ‘screening’; or ‘unknown’.

### Patient Characteristics

Antimicrobial prescription records for patients in which *S. aureus* was isolated, were obtained from seven Belgian health insurance funds (via IMA-AIM). Consumption data were categorized using the Anatomical Therapeutic Chemical (ATC) classification (World Health Organisation, WHO Collaborating Centre for Drug Statistics Methodology) up to four digits (e.g. J01C) and accordingly transformed into defined daily doses (DDD). Following additional patient characteristics were included in the analysis; age, sex, and admission to an acute, long term care (e.g. nursing homes), or other healthcare facility. Only observations prior to the moment of sampling (minimum one day) were considered for the inference analysis.

### Statistical Analysis

Univariate logistic regression was used to presumptively identify risk factors for oxacillin resistance *S. aureus* presence (MRSA). Single factors with a p-value <0.20 were retained for a stepwise forward multiple-factor analysis. Factors were recategorized due to analytical restrictions and conform the age groups applied by the Belgian IMA.

For all analyses, the significance level was set at α = 0.05. All tests were performed using the statistical software package STATA version 10.0 (Stata Corporation, College Station, TX, USA).

### Ethics Statement

None of the participants (nor next of kin, caretakers, or guardians on the behalf of the minors/children participants) provided an informed consent because data from laboratories and reimbursement organizations were encrypted by a third trusted party to ensure patient confidentiality. This consent procedure and the entire study protocol was approved by the *Sectorial committee of the Belgian Federal Social Security* as well as by the jointed ethical committee of the *Scientific Institute of Public Health* (WIV-ISP) and the *Veterinary and Agrochemical Research centre* (CODA-CERVA).

## Results

### Microbiology and Matching of the Records

Initially, a total of 92 117 samples (107 130 isolates; Table 1) were retrieved from the laboratories, from which 104 970 bacteria were retained originating from 44 365 patients after data cleaning and validation. For one entire laboratory, no link could be made between their microbiological data and the IMA records.

**Table 1:** Isolate distribution of the initially retrieved microbiological records from 17 Belgian laboratories (2005).

Matrix (sample type)	Sample population		SA Study sample	
	N	%	N	%
Sterile organs				
Blood	8 523	7.96	291	4.25
CSF	267	0.25	5	0.07
Other Aspiration	356	0.33	16	0.23
Respiratory tract				
URT: excluding Eye, Ear, Sinus	5 410	5.05	686	10.02
URT: Eye, Ear, Sinus	1 090	1.01	178	2.60
LRT: Sputum excluded	6 814	6.36	494	7.22
LRT: Sputum	7 578	7.07	628	9.18
Gastro-Intestinal tract				
GIT: faeces	1 422	1.32	36	0.53
GIT: faeces excluded	1 054	0.98	221	3.23
Uro-Genital Tract				
Urine	42 014	39.24	540	7.89
Urogenital tract urine excluded	6 996	6.53	957	13.98
Diverse				
Corpora aliena	2 015	1.89	81	1.18
Tissue samples/biopsies	2 165	2.02	132	1.93
SSTI (including pus)	17 309	16.16	2427	35.46
Surveillance/Screening	2 068	1.93	109	1.59
Unknown	2 022	1.88	43	0.63
Σ/Total	107 130	100.00	6 844	100.00

SA: *Staphylococcus aureus* strains samples, including only isolates for which oxacillin susceptibility test result was available; CSF: cerebrospinal fluid; URT: upper respiratory tract; LRT: lower respiratory tract: GIT: gastro-intestinal tract; SSTI: skin & soft tissue infections.

The most highest antimicrobial use (in DDD; total: 2 620 734 DDD) was reported in the J01C class of beta-lactams, penicillins (972 198 DDD; 37.1%) followed by J01M quinolones (419 972 DDD; 16.0%), J01X other antimicrobials (351 453 DDD; 13.4%) and J01D other beta-lactams (270 793 DDD; 10.3%). Amoxicilline & enzyme inhibitor (J01CR02; 680 278 DDD; 26.0%), nifurtoinol (J01XE02; 194 335 DDD; 7.4%), ciprofloxacine (J01MA02; 177 904 DDD; 6.8%) and cefuroxime (J01DC02; 172 838 DDD; 6.6%) were the drugs most frequently prescribed. (The susceptibility results of the total 15 442 *S. aureus* isolates are presented in Table S1).

After exclusion of mislabelled patients (e.g. dummy attributed by IMA for several reasons including foreign travellers not participating to the Belgian social security system), and retention of the index samples for which an oxacillin test result was available, a total of 6 844 *S. aureus* cases were analysed. The sample matrix distribution of the latter subset is included in Table 1, and demonstrates only a minority originated from screening samples (1.59%).

More detailed information on prescribed antimicrobial classes and molecules, as well as on the microbiological results can be found elsewhere in Dutch and French (http://www.nsih.be/download/AB/multicentrstudienov2008.pdf).

### Regression Analysis

A total of 6844 index-patients with *S. aureus* infection/colonization and known oxacillin susceptibility tests results were retained in a final logistic regression model. The ratio male/female was 3446/3398 with the number of isolates (n MRSA) by age as follows: 0–9y: 629 (55); 10–19y: 276 (25); 20–29y: 417 (43); 30–39y: 452 (76); 40–49y: 518 (97); 50–59y: 679 (187); 60–69∶806 (259); 70–79y: 1410 (568); 80–89y: 1328 (657); 90–99y: 324 (210); 100–104y: 5 (4). Of these index patients 17.5% (n = 1200) died in 2005. Within the latter group, 51.2% (n = 614) were MRSA positive whereas in the patients who survived 2005 only 28.1% (1568/5644) were found to have MRSA (unadjusted OR for death in case of MRSA = 2.68; 95% CI 2.36–3.05; p<0.01).

When comparing MRSA and MSSA individuals, factors univariately significantly associated with MRSA were as follows (Odd’s Ratio, 95% confidence interval): health care facility type (hospital: 1.64, 1.43–1.89; long term care facility: 8.67, 6.97–10.87; other facilities (e.g. military hospital, burning centre): 2.55, 1.71–3.80) compared to no admission ( = reference), female gender (1.12, 1.01–1.25)., antimicrobial consumption (minimum 1 day prior to sample date) in ambulant care (2.44, 2.10–2.85) and intramural (3.90, 3.30–4.61), total DDD (minimum 1 day prior to sample date) (OR 95% CI variable depending on categories; 0 ( = reference); 0–9 DDDs: 1.48–2.12; 9–24 DDDs: 2.27–3.22; 24–59 DDDs: 2.34–3.33; 59–1191.5 DDDs: 3.88–5.47), and age (0–14 years = reference; 14–55 years: 2.09, 1.58–2.75; 55–104 years: 7.42,5.75–9.57).

In the final retained multivariate model following factors were independently associated with MRSA colonization/infection: admission to a long term care settings; supply by a hospital pharmacy, age, and consumption of antimicrobial agent per DDD (Table 2).

**Table 2:** Multivariate model to identify risk factors (odd’s ratios, OR) for methicillin resistant *Staphylococcus aureus*.

Variable	N	Adjusted OR	(95%CI)	p-value
MRSA positive related to type of health care setting				
No admission	1527	1	–	
Acute hospital	4647	0.86	0.74–1.01	0.069
Nursing home (LTCF)	560	3.53	2.79–4.46	<0.001
Other setting	110	1.43	0.93–2.19	0.102
Localisation AB prescription prior to sampling (minimum 1 day)				
Absent	1519	1	–	
Ambulant	3706	0.91	0.73–1.14	0.425
In hospital	1619	1.62	1.30–2.01	<0.001
Amount of AB use prior to sampling				
per DDD		1.32	1.25–1.40	<0.001
Age category				
0–14	757	1	–	
15–54	1837	1.63	1.23–2.16	0.001
55–104	4250	4.32	3.32–5.63	<0.001

MRSA: methicillin resistant *Staphylococcus aureus*; CI: confidence interval; LTCF: long term care facility; AB: antimicrobial (antibiotic); DDD: defined daily dose.

## Discussion

The present study aimed to investigate the dose-relationship between antimicrobial consumption and resistance. The relationship is well studied at aggregated level [6], [7], but at the individual level large studies are scarce. We like to refer to a reflection by Grundmann and colleagues [8]. They have stated that ‘to address the burden of disease attributable to antibiotic resistance, comprehensive enrolment of patients is needed, with follow-up beyond hospital discharge and a pathogen-specific approach to inform health-care providers and the public about the importance of this health threat’. In this study, MRSA was chosen because of its clinical relevance and as well studied proxy for resistance in large study populations. Our findings confirmed the relationship between antibiotic consumption and MRSA as demonstrated in a systematic review and meta-analysis [3]. Here, in addition, we could quantify a dose-response effect at the single individual level in one multi-centre design. Their meta-analysis was based upon free available papers and found for adults (>16 years old) an increased risk for MRSA following antimicrobial therapy both in hospitals and in the community. During another recent meta-analysis it was shown that antimicrobial consumption in primary care is associated with resistance, but this was not the case for MRSA predominantly isolated from skin and soft tissue infections including those isolated in children [9]. Our results based on a multi-centre analysis covering all ages and different healthcare settings in one design, confirmed on the one hand these results. On the other hand, we were able to quantify the institutional and consumption patterns by one of the most robust units of measurement used worldwide, namely the defined daily dose [10]. Other statistical techniques, like time series analysis, might help to further identify a lag effect of certain antimicrobial compounds in relation to resistance. An important limitation of our study is that only individuals were included that have been undergoing a microbiological survey in the study period, and care should be given when extrapolating the findings to the entire Belgian patient population (see below).

We demonstrated that hospitalisation is not a risk factor for MRSA as such in acute care, although prescription of antimicrobials in hospitals clearly is. On the other hand, residency in a long term care facility was identified as a risk factor for MRSA in our study. A decline of MRSA in nursing homes from 19% in 2005 to 13% in 2010, along with a parallel decline in Belgian hospitals has been documented [11], [12]. Adapted antimicrobial stewardship programs will be needed to combat resistance in these facilities.

General practitioners (GP) can consult enclosed susceptibility results for empiric treatments of MRSA infections in the community. However, a substantial selection bias might overestimate the resistance situation in the field since, and as stated previously, only patients that underwent bacteriological examinations were included here. Studies [12] have demonstrated circa 40% of MRSA positive people are recurrent cases. Microbiological results are not requested routinely in ambulatory care and thus a selection bias of patients difficult to treat, or therapy failures might be overrepresented here [9]. Within the concerned patient population, we do feel on the other hand confident with the clinical importance. This is because in our analysis only a minority of samples (1.93%) were retrieved from screening samples or for surveillance purposes (Table 1).

Our results on antimicrobial drug resistance patterns cannot be extrapolated to the Belgian community as such. Since GPs will treat the majority of patients in ambulatory care empirically, the microbiology reported in this study will not be representative for *S. aureus* antibiograms in the community. This study and the conclusions only concern patients that underwent reimbursed microbiology. Although this bias might have favoured resistance in our isolates, the narrow OR range (1.25–1.40) and thus strong relationship between consumption of antimicrobial agents and MRSA provides a clear quantitative assessment which will help clinicians to improve their understanding of resistance. The clinical relevance can be further exemplified by the crude mortality found here (95% CI OR, 2.36–3.05), in line with earlier investigations [1], [13], [14]. We did not take into account comorbidities or other underlying conditions (e.g. invasive devices) that are likely to lower the attributable mortality of for resistance [1]. In our research, only age and type of healthcare setting were included as variables, and can be interpreted as a proxy for comorbidities and unfavourable conditions. Geographical variables and prescriber ID were also not used in this study. Inclusion of such variables in future studies might further improve the modelling.

Another weaknesses of the study is the exclusion of a substantial number of patients due to incompatibility of the original data, and the absence of molecular typing methods (e.g. multiplex PCR) to confirm the identification of MRSA. The variety in the applied phenotyping methodologies for identification (e.g. selective agar) and susceptibility testing over different laboratories is a limitation inherent to the chosen study design.

## Conclusions

The results of the present analysis confirm the importance of health care settings and age on the presence of MRSA. Clinicians should consider that antimicrobial consumption inherently bears an individual risk for their own patient, besides the resistance induction at the population level that has been known for a long time.

## Supporting Information

Table S1 Disk diffusion susceptibility profiles of 15 442 *Staphylococcus aureus* strains (n patients = 7309) retrieved from 16 Belgian clinical laboratories (2005).
(DOCX)
