# Supplementary material for: Risk Factors for Methicillin Resistant Staphylococcus aureus: A Multi-Laboratory Study
Source: PLoS One. 2014 Feb 26;9(2):e89579. doi: 10.1371/journal.pone.0089579 (PMC3935888; doi:10.1371/journal.pone.0089579)
Supplement: Table S1 — Disk diffusion susceptibility profiles of 15 442 Staphylococcus aureus strains (n patients = 7309) retrieved from 16 Belgian clinical laboratories (2005). (DOCX) [file pone.0089579.s001.docx]

Table S1. Disk diffusion susceptibility profiles of 15 442 *Staphylococcus aureus* strains (n patients = 7309) retrieved from 16 Belgian clinical laboratories (2005).

| Antibiotic | n isolates | % tested | % Resistance (Non susceptibility + Intermediate) |
| --- | --- | --- | --- |
| Beta-lactams | | | |
| Penicillin | 11 686 | 75.68 | 91.59 |
| Ampicillin/amoxicillin | 6 813 | 44.12 | 91.93 |
| Amoxicillin-clavulanic acid | 4 074 | 26.38 | 27.66 |
| Cefalotin (group) | 2 267 | 14.68 | 24.57 |
| Cefazolin | 1 365 | 8.84 | 27.77 |
| Cefotaxim (ceftriaxone) | 1 169 | 7.57 | 37.90 |
| Cefoxitin | 3 177 | 20.57 | 51.46 |
| Cefuroxim | 1 277 | 8.27 | 33.83 |
| Oxacillin | 14 373 | 93.08 | 35.72 |
| Imipenem | 2 576 | 16.68 | 26.79 |
| Macrolides-lincosamides-streptogramines (MLS_B_) | | | |
| Erythromycin | 13 053 | 84.53 | 36.11 |
| Azithromycin | 1 803 | 11.68 | 28.90 |
| Clarithromycin | 750 | 4.86 | 28.80 |
| Clindamycin | 13 303 | 86.15 | 26.69 |
| Quinipristin-dalfopristin | 3 484 | 22.56 | 0.98 |
| Tetracyclines | | | |
| Tetracycline | 5 944 | 38.49 | 12.15 |
| Doxycycline | 5 817 | 37.67 | 19.40 |
| Minocycline | 1 913 | 12.39 | 1.25 |
| Potentiated sulphonamides (cotrimoxazole) | | | |
| Trimethroprim - sulphonamiden | 9 461 | 61.27 | 39.78 |
| Fluoroquinolones | | | |
| Norfloxacin | 5 298 | 34.31 | 34.81 |
| Ciprofloxacin | 8 659 | 56.07 | 40.65 |
| Ofloxacine | 1 789 | 11.59 | 30.07 |
| Levofloxacin | 6 014 | 38.95 | 36.12 |
| Aminoglycosides | | | |
| Gentamicin | 12 704 | 82.27 | 2.93 |
| Kanamycin | 698 | 4.52 | 16.62 |
| Tobramycin | 4 923 | 31.88 | 14.00 |
| Other | | | |
| Nitrofurantoin | 5 955 | 38.56 | 4.45 |
| Fosfomycin | 4 229 | 27.39 | 5.70 |
| Fusidicic acid | 8 558 | 55.42 | 6.10 |
| Mupirocin | 3 556 | 23.03 | 2.81 |
| Novobiocin | 788 | 5.10 | 0.25 |
| Rifampicin | 7 561 | 48.96 | 1.48 |
| Teicoplanin | 8 201 | 53.11 | 0.10 |
